# Supplementary material for: Understanding the Dosage-Dependent Role of Dicer1 in Thyroid Tumorigenesis
Source: Int J Mol Sci. 2024 Oct 4;25(19):10701. doi: 10.3390/ijms251910701 (PMC11476720; doi:10.3390/ijms251910701)
Supplement: Supplementary file 1 [file ijms-25-10701-s001.zip › ijms-3156242-supplementary.pdf]

**SUPPLEMENTARY DATA**

| mRNA | Forward | Reverse |
|---|---|---|
| *ICAM1* | 5’ - AGAGACCCCGTTGCCTAAAA - 3’ | 5’ - AGTACACGGTGAGGAAGGTT - 3’ |
| *PNMA2* | 5’ - AGGTGGGTGATGTCTTCCTG - 3’ | 5’ – ATAGTTGAGGCACCTGGGAC - 3’ |
| *ZNF28* | 5’ - TTTGCAATGTCCCTGTGTGG - 3’ | 5’ - CTCACTGCAACCTCCACTTC - 3’ |
| *EPHA5* | 5’ - TCTCTGTATACCGGCCCATC- 3’ | 5’ - GCAGGCCCAAGTTTGATGAA- 3’ |
| *ZNF468* | 5’ - GTCTGTGCAATGTCCCTGTG - 3’ | 5’ - TCCCGGCTAAAGTACAGTGG- 3’ |
| *PPP1R3C* | 5’ - GCCCGACACTAGGATTCTCA - 3’ | 5’ - CCTCTCAATGGTTGTGCACA - 3’ |
| *SMIM10* | 5’ - TGAACCAGGTAGCCAACAGA- 3’ | 5’ - TCTCAACTCTTGCTTCCCCA- 3’ |
| *LINC01234* | 5’ - GAGACAGACAGCAAGAGA - 3’ | 5’ - ACCACACCTGAGATAAGC - 3’ |
| *CASP10* | 5’ - TGATGTCTACCGCAGCAGAA- 3’ | 5’ - GCAGGAACAGGCCATTTTCA - 3’ |
| *P16* | 5’ - CACCAGAGGCAGTAACCATG - 3' | 5’ - GTTTCCCGAGGTTTCTCAGAG - 3’ |
| *P21* | 5’ - TGTCACTGTCTTGTACCCTTG - 3' | 5’ - GGCGTTTGGAGTGGTAGAA - 3' |
| *P27* | 5’ - TCTGAGGACACGCATTTGG - 3' | 5’ - TGTTCTGTTGGCTCTTTTGTT - 3' |
| *DICER1* | 5’-TTAACCTTTTGGTGTTTGATGAGTGT- 3' | 5’ - GCGAGGACATGATGGACAATT -3' |
| *NEDD8* | 5’-TGACCGGAAAGGAGATTGAGAT - 3’ | 5’ - CCTCCACACGCTCCTTGATT - 3’ |
| *TTC1* | 5’-CGGAGAAGCTGTGAGGTTCTTTA - 3’ | 5’ - TCCTCTGGAACCCCACAGTT - 3’ |

**Table S1. Sequence of the primers used for RT-qPCR analysis**

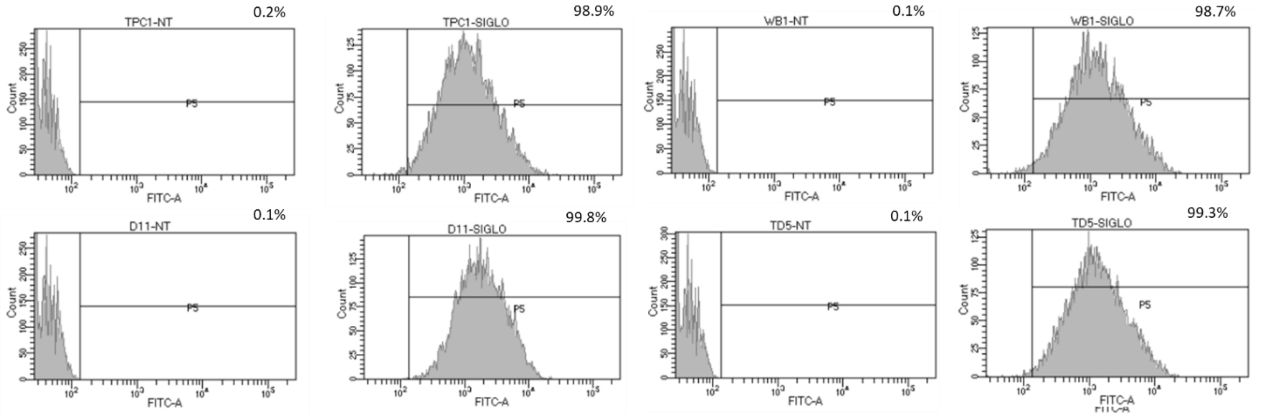


**Figure S1. Transfection efficiency is higher than 98% in TPC1 and derived *Dicer1* (+/-) cell lines.** Transfection efficiency was assessed in TPC1, D11, WB1 and TD5 cells through flow cytometry using a fluorescent mimic SIGLO (5nM), in non-transfected cells (NT) or in cells transfected with the fluorescent mimic SIGLO (SIGLO).

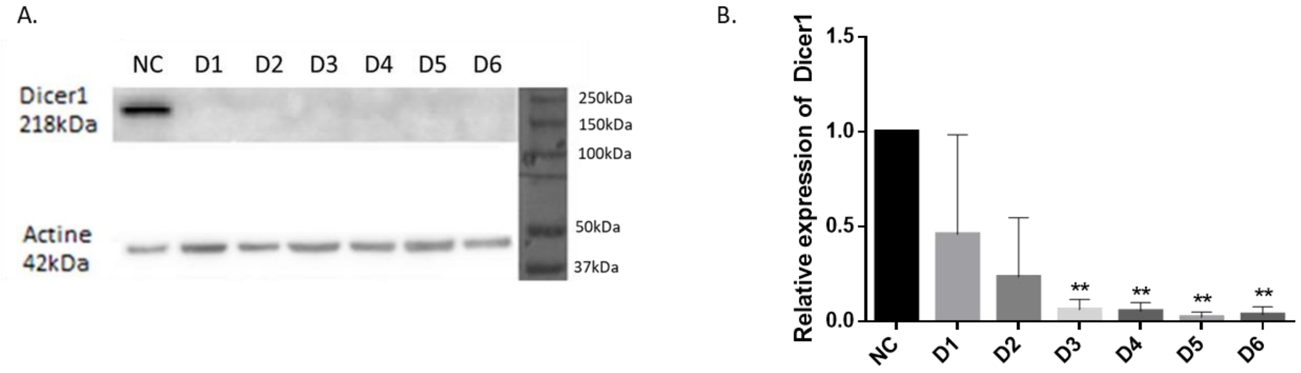


**Figure S2. Dicer1 protein is undetectable 24 hours after siRNA transfection and remains absent up to 6 days after transfection. (A)** Western Blot analysis of Dicer1 protein expression in TPC1 after transfection**.** Cell cultures were transfected with negative control (NC) or Dicer1 siRNA and samples were collected at regular intervals over the following days: one (D1), two (D2), three (D3), four (D4), five (D5) and six days after transfection (D6). **(B)** Western Blot quantification of Dicer1 protein expression (n=3). The columns represent the mean values, and the error bars indicate the standard deviation (mean ± SD). Statistically significant differences were determined using Kruskal-Wallis test. ** $p < 0.01$.

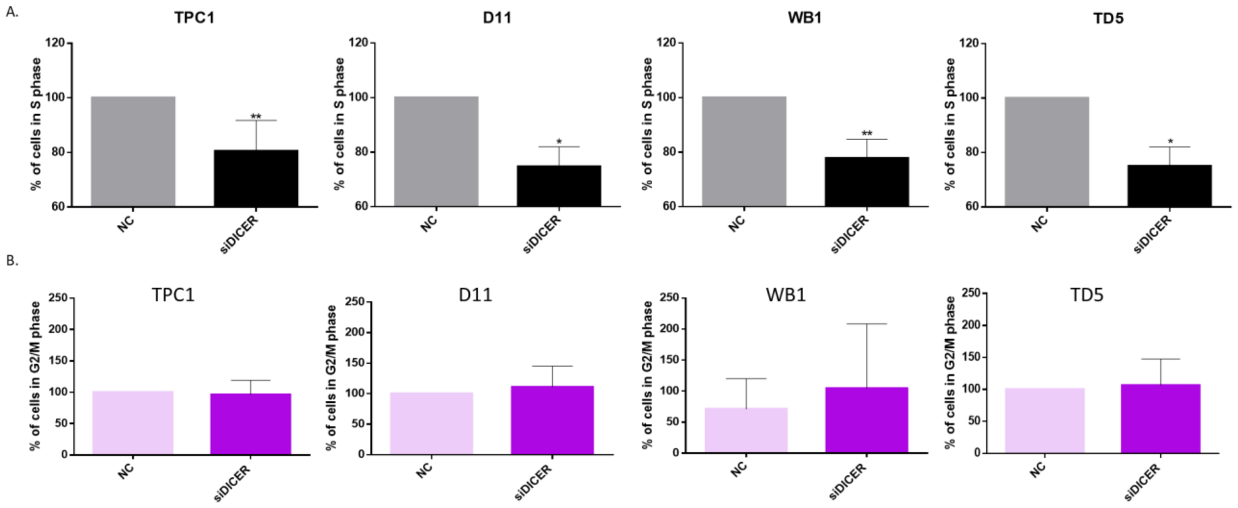


**Figure S3. Total *Dicer1* loss inhibits proliferation but does not modify the percentage of cells in G2 and M phases.** Cells were intracellularly stained with BrdU Staining Kit for Flow Cytometry. The percentage of positive or negative cells was determined by flow cytometry. **(A)** Quantification of cells in S-phase in cells transfected with the negative control (NC) or *Dicer1* siRNA (siDICER) in

TPC1 (n=6), D11 (n=4), WB1 (n=6), and TD5 (n=4) cell lines. **(B)** Quantification of cells in the G2/M phases of the cell cycle in TPC1 (n=5), D11 (n=5), WB1 (n=5), and TD5 (n=7) cell lines following negative control (NC) or *Dicer1* siRNA (siDICER) transfection. Data were normalized to the NC. The columns represent the mean values, and the error bars indicate the standard deviation (mean ± SD). Statistically significant differences were determined using Mann-Whitney t-test. * $p < 0.05$ and ** $p < 0.01$.

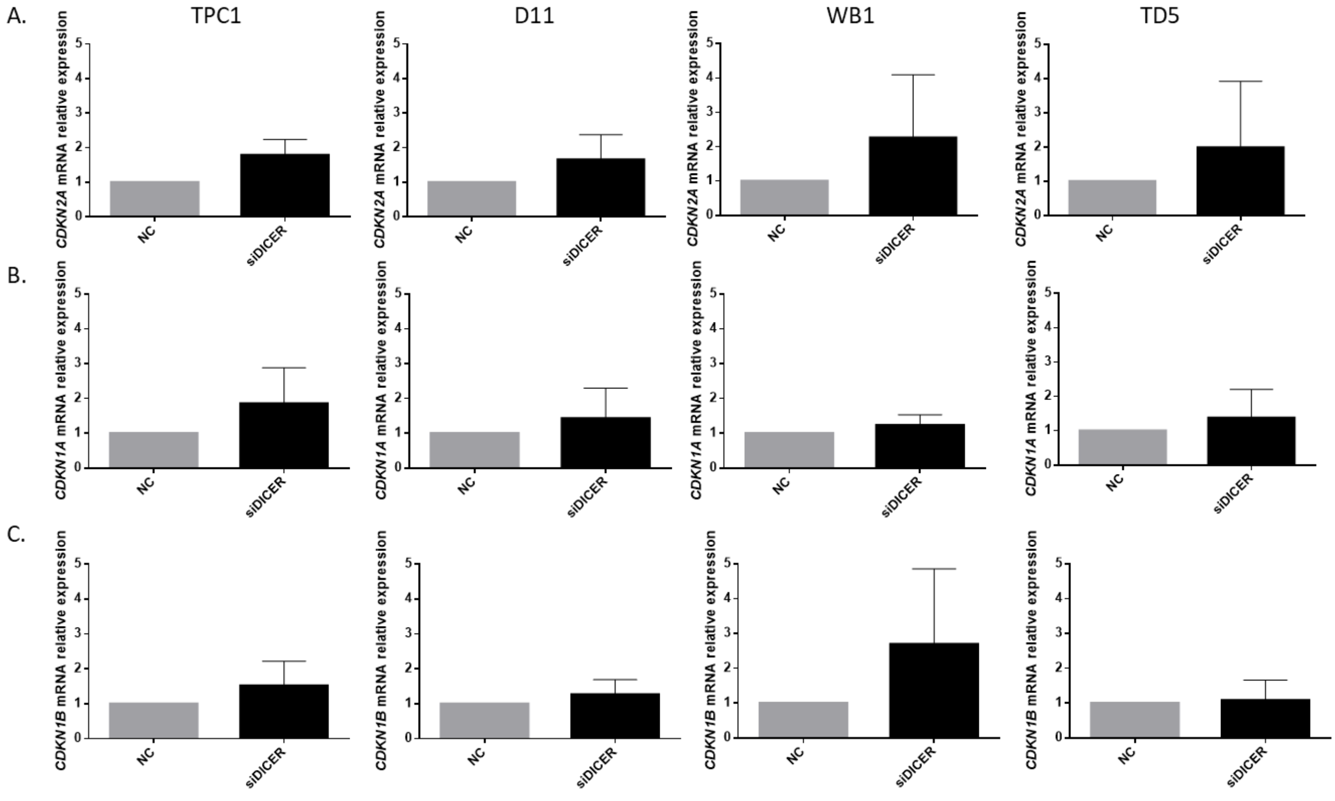


**Figure S4. mRNA expression levels of *p16 (CDKN2A), p21 (CDKN1A)* and *p27 (CDKN1B)*.** mRNA expression was analyzed by RT-qPCR in cells transfected with the negative control (NC) or with *Dicer1* siRNA (siDICER) four days after-transfection in the TPC1 (n=7), D11 (n=5), WB1 (n=4) and TD5 (n=4) cell lines. The columns represent the mean values, and the error bars indicate the standard deviation (mean ± SD). Statistically significant differences were determined using Mann-Whitney t-test.

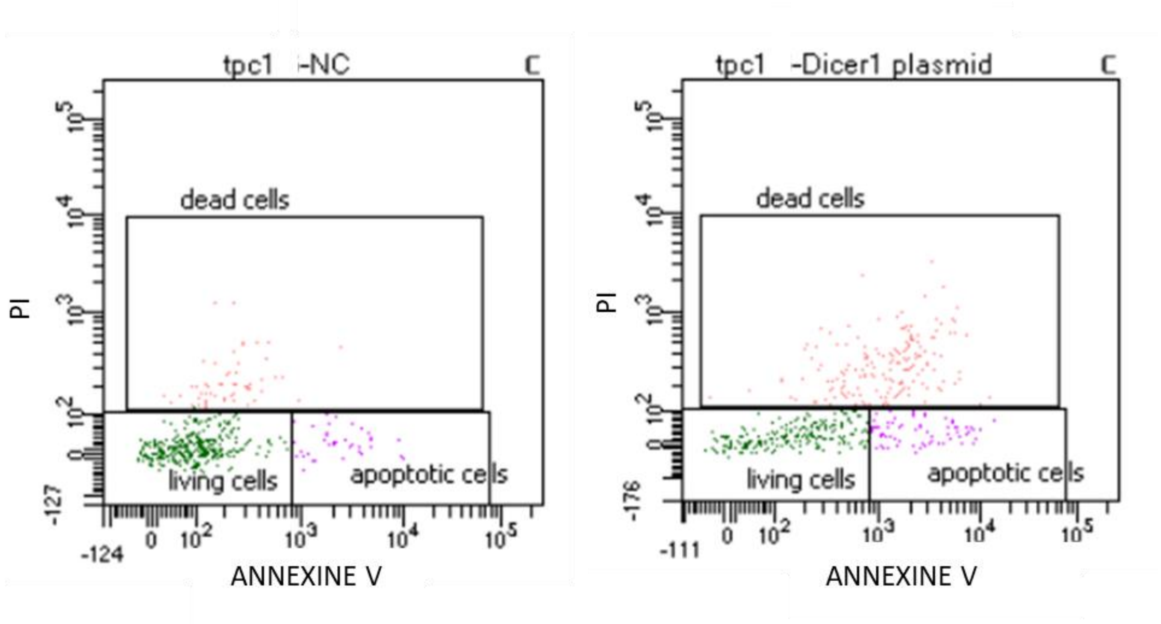


**Figure S5. Annexin V/PI analysis demonstrated a significant increase in apoptosis following *Dicer1* knockdown by siRNA.** The PI/Annexin V test is used to detect cell apoptosis by distinguishing live, apoptotic, and dead cells based on the binding of Annexin V to phosphatidylserine and PI staining of membrane-compromised cells. For each condition, negative control (NC) at left and *Dicer1* plasmid transfection at right, cells were stained using the Dead Cell Apoptosis Kit with Annexin V-FITC and Propidium Iodide (PI) for flow cytometry (V13242, ThermoFisher).

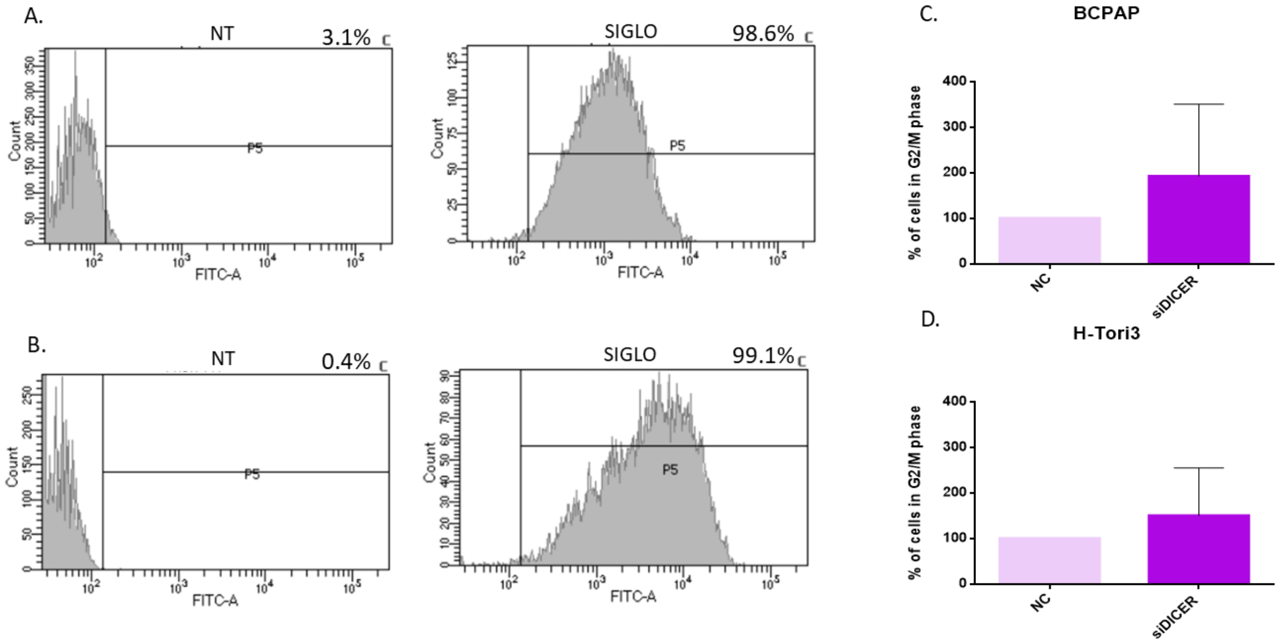


**Figure S6. (A, B) Transfection efficiency is higher than 98% in BCPAP and H-Tori3 cell lines.** Transfection efficiency was assessed in BCPAP (A) and H-Tori3 (B) cells through flow cytometry using a fluorescent mimic SIGLO (5mM), in non-transfected cells (NT) or in cells transfected with the fluorescent mimic SIGLO. **(C, D) Total loss of *Dicer1* does not modify the percentage of cells in G2 and M phases.** Quantification of cells in the G2/M phases of the cell cycle in BCPAP (C, n=5) and H-Tori3 (D, n=8) following negative control (NC) or *Dicer1* siRNA (siDICER) transfection. Data were normalized to the NC. The columns represent the mean values, and the error bars indicate the standard deviation (mean ± SD). Statistically significant differences were determined using Mann-Whitney t-test.

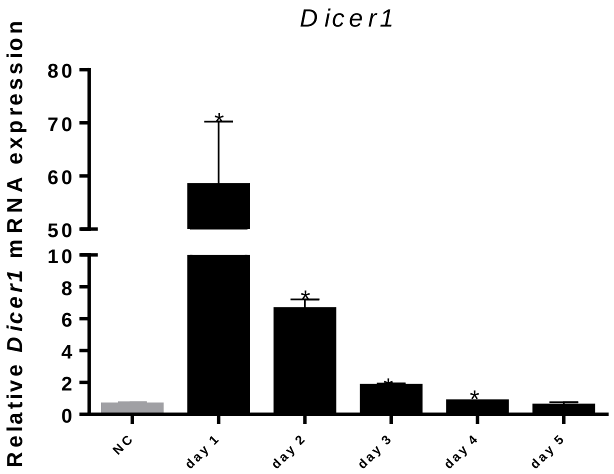


**Figure S7. *Dicer1* mRNA is highly expressed during four days following *Dicer1* plasmid transfection.** (A) qPCR analysis of *Dicer1* expression in TPC1 after transfection**.** Cell cultures were transfected with negative control (NC) or Dicer1 plasmid and samples were collected at regular intervals over the following days: one (D1), two (D2), three (D3), four (D4) and five (D5) after transfection. The columns represent the mean values, and the error bars indicate the standard deviation (mean ± SD). Statistically significant differences were determined using Mann-Whitney test * $p < 0.05$

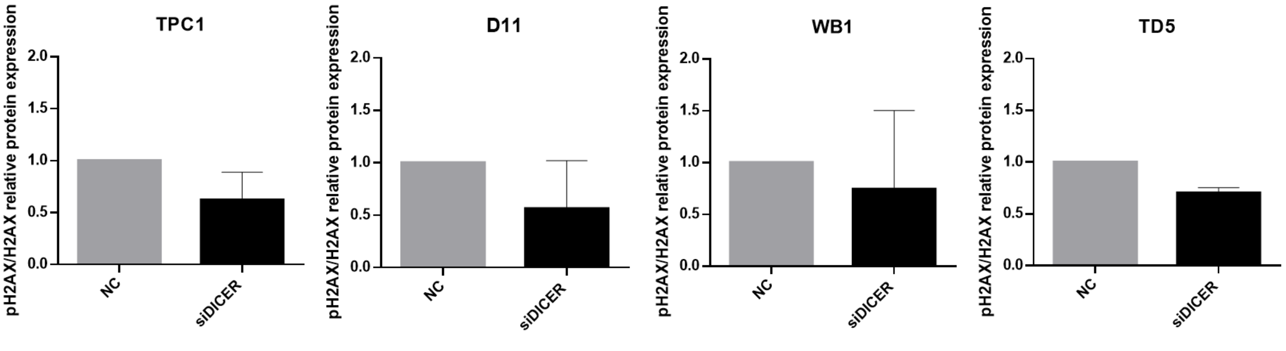


**Figure S8. pH2AX levels were unaltered following *Dicer1* siRNA transfection.** Western Blot analysis of phospho-H2AX and H2AX protein expression in TPC1 (n=3), D11 (n=3), WB1 (n=3) and TD5 (n=2) cell lines following negative control (NC) or *Dicer1* siRNA (siDICER) transfection. Phosphorylated histone H2AX (pH2AX) expression was normalized to H2AX expression and data were normalized to the NC. The columns represent the mean values, and the error bars indicate

the standard deviation (mean ± SD). Statistically significant differences were determined using Mann-Whitney t-test.
